# Ambient Influenza and Avian Influenza Virus during Dust Storm Days and Background Days

**DOI:** 10.1289/ehp.0901782

**Published:** 2010-04-30

**Authors:** Pei-Shih Chen, Feng Ta Tsai, Chien Kun Lin, Chun-Yuh Yang, Chang-Chuan Chan, Chea-Yuan Young, Chien-Hung Lee

**Affiliations:** 1 Department of Public Health, College of Health Science, Kaohsiung Medical University, Kaohsiung City, Taiwan; 2 Institute of Occupational Medicine and Industrial Hygiene, College of Public Health, National Taiwan University, Taipei City, Taiwan; 3 Department of Natural Resource, College of Agriculture, Chinese Culture University, Taipei City, Taiwan

**Keywords:** ambient virus, avian influenza virus, bioaerosol, dust storm, infectious bioaerosol, influenza virus, quantification, real-time qPCR

## Abstract

**Background:**

The spread of influenza and highly pathogenic avian influenza (H5N1) presents a significant threat to human health. Avian influenza outbreaks in downwind areas of Asian dust storms (ADS) suggest that viruses might be transported by dust storms.

**Objectives:**

We developed a technique to measure ambient influenza and avian influenza viruses. We then used this technique to measure concentrations of these viruses on ADS days and background days, and to assess the relationships between ambient influenza and avian influenza viruses, and air pollutants.

**Methods:**

A high-volume air sampler was used in parallel with a filter cassette to evaluate spiked samples and unspiked samples. Then, air samples were monitored during ADS seasons using a filter cassette coupled with a real-time quantitative polymerase chain reaction (qPCR) assay. Air samples were monitored during ADS season (1 January to 31 May 2006).

**Results:**

We successfully quantified ambient influenza virus using the filtration/real-time qPCR method during ADS days and background days. To our knowledge, this is the first report describing the concentration of influenza virus in ambient air. In both the spiked and unspiked samples, the concentration of influenza virus sampled using the filter cassette was higher than that using the high-volume sampler. The concentration of ambient influenza A virus was significantly higher during the ADS days than during the background days.

**Conclusions:**

Our data imply the possibility of long-range transport of influenza virus.

The spread of highly pathogenic avian influenza (H5N1) into Asia, Europe, and even Africa has strongly affected the poultry industry and presents a significant threat to human health. To date, 363 human cases of avian influenza (61% of them fatal) have been officially reported by the World Health Organization (2008). In 2003, the rapid spread of severe acute respiratory syndrome (SARS) to Asia, North America, Europe, and Australia during the first two quarters of the year illustrated the speed at which influenza and avian influenza pandemics can spread across the world. Influenza and avian influenza outbreaks are expected to be much harder to control than SARS because, in contrast with SARS, people infected with influenza are contagious before the onset of case-defining symptoms (Koh et al. 2008). Therefore, it is important to understand possible transmission pathways between countries in preparation for influenza or avian influenza pandemics.

How the highly pathogenic H5N1 avian influenza has spread between countries has been extensively debated. In a previous study, Kilpatrick et al. (2006) integrated data on phylogenic relationships of virus isolates, poultry and wild bird trade, and migratory bird movements to determine the pathway for the introduction of H5N1 into each of 52 countries. Their results demonstrated that 9 of 21 H5N1 inductions into countries in Asia were most likely through poultry, and 3 of 21 were through migrating birds. However, H5N1 outbreaks in South Korea and Japan were not consistent with either reported poultry trade or the timing and direction of migratory bird travel during the month of outbreak, suggesting that other factors led to these introduction events.

Avian influenza outbreaks in Japan and South Korea, which, like Taiwan, include areas that are downwind of Asian dust storms (ADS), occurred during the ADS season, according to reports from the World Organization for Animal Health (OIE 2006). With increasing evidence from epidemiological studies, increased health effects, including respiratory diseases, during ADS days in downwind areas have recently drawn much attention (Bell et al. 2008; Chan et al. 2008; Chang et al. 2006; Chen et al. 2004; Chen and Yang 2005; Yang et al. 2005a, 2005b). In addition, several researchers have reported that the presence of desert dust in the atmosphere is associated with increased concentrations of cultivable bacteria, cultivable fungi, and fungal spores during ADS that affected air quality in downwind areas relative to background levels or days with clear atmospheric conditions (Brown et al. 1935; Fulton 1966; Griffin 2007; Griffin et al. 2001, 2003, 2006, 2007; Ho et al. 2005; Kellogg et al. 2004; Kwaasi et al. 1998; Prospero 1999; Schlesinger et al. 2006), which suggests that long-range transport of air pollutants contributed to local bioaerosol levels. However, viral concentrations in ambient air have not been researched in association with ADS, possibly due to a lack of sampling and analytical methods.

In a previous study, we successfully quantified airborne influenza and avian influenza virus levels in a live-animal (“wet”) poultry market using a filtration/real-time quantitative polymerase chain reaction (qPCR) method (Chen et al. 2009), thus demonstrating that this quantitative technique could provide information that could be used to study the possible long-range transport of influenza and avian influenza virus. However, higher sampling rates or longer sampling times may be necessary to measure extremely low virus concentrations in ambient air, and both of these approaches have the potential to injure or destroy viruses and thus inhibit detection. To address these concerns and identify an assay suitable for quantifying airborne viruses, we compared the performance of a high-volume air sampler and that of a filter cassette for evaluation of ambient influenza and avian influenza virus. Next, we determined concentrations of ambient influenza/avian influenza virus during ADS days and background days using the more sensitive method, and evaluated relations between environmental parameters and ambient influenza virus levels.

## Materials and Methods

### Comparison of samplers

Airborne influenza and avian influenza viruses in a wet poultry market were successfully collected on 0.2-μm-pore polytetrafluoroethylene (PTFE; Teflon) membrane filters in disposable plastic cassettes (37 mm) as previously described (Chen et al. 2009). For comparison to determine an assay suitable for quantifying airborne viruses, we evaluated a high-volume air sampler, MFC-PM10 (model TE-6070; Tisch Environmental, Inc., Cleves, OH, USA), at a sampling rate of 1,100 L/min, in parallel with a PTFE cassette at a sampling rate of 20 L/ min for both spiked and unspiked samples at the Wan-Li air monitoring station near the northern tip of Taiwan. For the spiked sampling, we applied a medium that contained A/ Hiroshima/52/2005 H3N2 virus onto clean filters (Pall Corp., New York, NY, USA). Then, air was sampled through the spiked filter for a 24-hr sampling period on 5 randomly chosen days (5 sets, *n* = 10). Air for unspiked sampling was sampled through a clean filter for a 24-hr sampling period on 13 randomly chosen days (13 sets, *n* = 26).

### Ambient influenza and avian influenza virus collection

Before the sampling, filters and support pads were autoclaved, and the plastic cassettes were sterilized with ethylene oxide. The samples were then transported at 4°C to our laboratory (Kaohsiung City, Taiwan, Republic of China) within 1 day. For quality control, trip blank and field blank controls were also evaluated. Results confirmed no detectable influenza virus RNA in either trip blanks or field blank controls (data not shown). In addition, side-by-side duplicate field samples yielded comparable results (with relative difference of 11%). Air samples were monitored during the ADS season (1 January to 31 May) in 2006 at two air monitoring stations run by the Taiwan Environmental Protection Administration (TEPA): Wan-Li (25°17′ N, 121°32′ E) in Shi-Men Township, a rural area (population density of 227/km^2^), and Shin-Jhuang (25°02′ N, 121°26′ E) in Shin-Jhuang City, an urban area (population density of 19,816/km^2^) (Wu et al. 2008). The Wan-Li station is located in a remote area near the northern tip of Taiwan that is upwind of Taipei during northeastern monsoons. The Shin-Jhuang station is located in Shin-Jhuang City, an important business and industrial center in Taipei County, close to two major highways that have heavy traffic (Wu et al. 2008).

### ADS in Taiwan

When significant ADS episodes originating in the deserts of Mongolia and western China were detected as yellow dust at ground observation stations in China, TEPA obtained real-time information through cross-country cooperation (Chan et al. 2008). Satellite images provided by the Taiwan Central Weather Bureau from the Moderate Resolution Imaging Spectroradiometer (MODIS) onboard the *Terra* or *Aqua* satellite were also used to track the ADS paths in East Asia. In addition, prediction models from Japan (http://www.jma.go.jp/jp/kosa/index.html), Korea (http://web.kma.go.kr/eng/asi/asi_02_04.jsp), and the United States (http://www.nrlmry.navy.mil/aerosol/index_shortcuts.html) were also used by TEPA to predict ADS trajectories. In this study, we collected 24-hr air samples beginning 12 hr before the predicted onset of each ADS episode. Because episodes occur during several days, we collected a total of ten 24-hr samples for each episode. Influenza virus was analyzed on days reported as ADS days according to TEPA (http://www.atmos.pccu.edu.tw/duststorm/database/database.htm). Sampling days after the end of each ADS episode were classified as representative background days. Trajectories of each ADS were tracked by satellite images, and TEPA predictions were confirmed using the U.S. National Oceanic and Atmospheric Administration (NOAA) Air Resources Laboratory Hybrid Single Particle Lagrangian Integrated Trajectory (HYSPLIT) model (http://www.arl.noaa.gov/hysplit-bin/trajtype.pl?runtype=archive) and global wind data from NOAA’s National Centers for Environmental Prediction Reanalysis data sets (http://ready.arl.noaa.gov/READYcmet.php). Air quality trends measured by three types of air monitoring stations in northern Taiwan were used to define the beginning and end of each ADS episode in Taipei. In addition, wind trajectories on both episode days and background days were confirmed using HYSPLIT back-trajectories. Concentrations of ambient influenza virus were expressed as copies of target cDNA/m^3^ air (copies/m^3^) per day.

### Viral genomic RNA isolation and realtime qPCR assay

Viral genomic RNA of influenza virus in the filters was isolated and analyzed as described previously (Chen et al. 2009). The commercially available QIAamp Viral RNA Mini Kit (Qiagen GmbH, Hilden, Germany) was used to isolate RNA. The procedure followed manufacturer’s recommendation, except that in step 2 in our study, “the sampled Teflon filter was folded into quadrants with virus inside and then placed upside down into the buffer AVL-carrier RNA in the 1.5-mL microcentrifuge tube” (Chen et al. 2009). The viral RNA was stored at −80°C until analysis within 1 month.

Table 1 shows the primers and probes used to amplify and identify influenza A virus and avian influenza (A/H5) virus. The primers and probe for influenza A virus target the matrix protein gene present in all types of influenza A. Primers and probes for A/H5 targeted conserved regions of North American H5 influenza viruses (Spackman et al. 2002; van Elden et al. 2001). Specificity was 100% for both influenza A and A/H5 viruses (Spackman et al. 2002; van Elden et al. 2001). In our study, samples were first analyzed for influenza A virus. Only those positive samples were then analyzed for A/H5.

Amplification and detection were performed using an ABI Prism 7500 sequence detection system (Applied Biosystems, Foster City, CA, USA) with a TaqMan One-step Reverse Transcriptase PCR Master Mix Reagents Kit (Applied Biosystems) with 5 μL viral RNA solution in an end volume of 25 μL as described previously (Chen et al. 2009). All samples analyzed using the real-time qPCR were done in triplicate. All manipulations of samples were performed in a biological safety cabinet.

Standard curves were derived as described in detail by Chen et al. (2009). In brief, the calibration curve was linear for 7 orders of magnitude with *r* > 0.988, and the detection limit of the filter/real-time qPCR method was 0.8 copy/m^3^ and 1.23 copies/m^3^ for influenza A and A/H5 viruses, respectively (Chen et al. 2009). The standard curve, positive controls, and negative controls were analyzed in triplicate for each run.

### Inhibitory effect

Cosampled compounds may inhibit amplification assays of environmental samples (Alvarez et al. 1995). Alvarez et al. (1995) reported that 10^3^ to 10^4^ colonyforming units (CFU) per cubic meter bacterial and fungal bioaerosols inhibited PCR amplification, whereas a 1/10 dilution of these samples did not. In the present study, all samples were analyzed simultaneously using 1, 1/10, 1/100, and 1/1,000 dilutions. Positive samples were those in which cDNA was quantified in any diluted solution. Then, the true concentration was obtained by multiplying the detected concentration by diluted factor (Chen et al. 2009). We classified samples as inhibitory if they were initially negative and then positive after dilution. We defined the inhibitory rate as the number of inhibitory samples divided by the number of positive samples.

### Environmental parameters

Hourly data for air pollution and meteorological parameters for the study period (1 January to 31 May 2006) were provided by the TEPA. Environmental parameters subjected to statistical analysis included concentrations of particulate matter (PM) with aerodynamic diameter ≤ 10 μm (PM_10_; micrograms per cubic meter), PM with aerodynamic diameter ≤ 2.5 μm (PM_2.5_; micrograms per cubic meter), nitrogen monoxide and nitrogen dioxide (NO_x_; micrograms per cubic meter), ozone (O_3_; parts per billion), sulfur dioxide (SO_2_; parts per billion), carbon monoxide (CO; parts per million), temperature, relative humidity (RH; percent), and rainfall.

### Statistical methods

Statistical analyses were performed using SigmaPlot for Windows (version 3.06; SPSS Inc., Chicago, IL, USA). The Mann-Whitney *U*-test was used to evaluate the difference between samplers and to estimate the impact of ADS on ambient influenza virus and on environmental factors. The chi-square test and Fisher exact test were used to evaluate the differences in positive rates and inhibitory rates between ADS days and background days. We used the Spearman correlation to evaluate relations between ambient influenza virus and environmental factors. Significance was accepted at *p* < 0.05.

## Results

### Sampler comparison

Table 2 summarizes measured concentrations of influenza virus in 24-hr samples using a high-volume sampler and a PTFE cassette in parallel for both spiked samples (5 sets, *n* = 10) and unspiked samples (13 sets, *n* = 26) at the Wan-Li air monitoring station. The average concentrations of influenza virus measured from both spiked and unspiked samples were higher when sampled with the PTFE cassette than with the high-volume sampler, although the differences were not statistically significant. In addition, the overall inhibitory rate was higher for the high-volume sampler than for the PTFE cassette (Table 2).

### Ambient influenza virus and ADS events

A total of six ADS episodes affected Taiwan from 1 January to 31 May 2006; a total of 24 days were classified as ADS days: three episodes affecting 5 days and three episodes affecting 3 days. We used two 24-hr samples taken 2 days after the end of each ADS episode (on days 8 and 9 after the first measurement before the predicted onset of three ADS episodes and on days 6 and 7 after the first measurement before the predicted onset of another three ADS episodes), except two lost samples, as background days (a total of 10 days). Table 3 shows the descriptive statistics for airborne influenza A virus and A/H5 during ADS days and background days at air monitoring stations in Taiwan.

For influenza A virus, both the positive rate (the number of positive samples divided by the number of all samples) and mean concentration were higher during ADS episodes than during background days at both monitoring stations (Table 3), with a significant difference in mean concentrations observed at the Wan-Li station on the northern tip of Taiwan (*p* < 0.05, Mann-Whitney *U*-test). Inhibitory rates for influenza A virus were higher during ADS days than during background days. For A/H5, only 3 of 68 samples were positive, with concentrations in the range of 2–25 copies/m^3^. All three of these positive samples were collected during episode days at the Wan-Li station.

**Figure 1:**
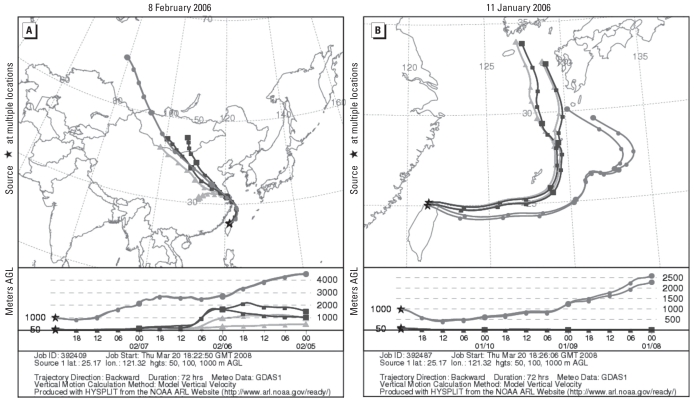
HYSPLIT back-trajectories of air masses arriving at the Wan-Li air monitoring station in Taiwan during the ADS period (*A*) and background days (*B*). Plots show 3-day air mass back-trajectories on 8 February 2006 (*A*) and 11 January 2006 (*B*). Abbreviations: AGL, above ground level; GDAS, Global Data Assimilation System (http://www.arl.noaa.gov/gdas1.php).

PM_10_, PM_2.5_, and CO concentrations were significantly higher (*p* < 0.05) during ADS days than during background days, whereas temperatures were significantly lower (*p* < 0.05; Table 4). No other environmental factors were significantly associated with the ADS episodes during the study period. HYSPLIT back-trajectories indicated that the trajectories of all positive samples collected during ADS periods were from mainland China (Figure 1A), whereas those collected during background days were not (Figure 1B).

### Associations between ambient influenza virus and environmental parameters

At the Wan-Li station, PM_10_ and PM_2.5_ concentrations were negatively correlated with ambient influenza A virus on both ADS days and background days, but only the correlation with PM_10_ on background days was significant (Table 5). In addition, mean concentrations of PM_10_ among samples positive for influenza A virus collected on ADS days and background days (43.35 μg/m^3^ and 20.33 μg/m^3^, respectively) were lower than PM_10_ concentrations among negative samples collected on ADS days and background days (53.82 μg/m^3^ and 37.19 μg/m^3^; *p*-values for both comparisons = 0.053). At the Shin-Jhuang station, PM_10_, PM_2.5_, NO_x_, SO_2_, and CO were all inversely correlated with ambient influenza virus A concentrations on background days but not on ADS days, but only the correlation with SO_2_ was significant (Table 5). The concentration of SO_2_ among samples positive for influenza A virus collected at the Shin-Jhuang station on background days (2.85 ppb) was significantly lower than that among negative samples (8.65 ppb, *p*-value 0.037). When we pooled samples from Wan-Li and Shin-Jhuang stations, ambient influenza A virus was significantly negatively correlated with PM_10_ and PM_2.5_ on background days (Table 5), and mean PM_10_ and PM_2.5_ concentrations were also significantly lower among samples positive for influenza A virus (22.86 μg/m^3^ and 10.85 μg/m^3^) than among negative samples (46.88 μg/m^3^ and 26.94 μg/m^3^; *p*-values for the difference in PM_10_ and PM_2.5_ concentrations of 0.010 and 0.013, respectively).

## Discussion

In this study, we successfully quantified ambient influenza virus using filtration/real-time qPCR. To our knowledge, this is the first report describing concentrations of influenza virus in ambient air. In previous studies, airborne infectious viruses have been detected using filtration coupled with a PCR-based method in indoor environments with high virus concentrations (e.g., hospitals and offices), including varicella-zoster virus, human cytomegalovirus, respiratory syncytial virus, acute respiratory syndrome coronavirus, and rhino-virus (Aintablian et al. 1998; McCluskey et al. 1996; Myatt et al. 2004; Sawyer et al. 1994; Tsai et al. 2006). In those studies, airborne viruses were only qualitatively or semiquantitatively detected, involving only positive or negative responses in a narrow dynamic range (< 4 orders of magnitude), and no concentration profiles were reported.

Airborne influenza viruses were successfully quantified in hospitals and wet poultry markets in two recent studies (Blachere et al. 2009; Chen et al. 2009). Because the use of a high-volume sampler would increase the total amount of virus collected in a given sample, enhancing detection sensitivity, we compared the performance of a high-volume sampler with that of a PTFE cassette. Our results (Table 2) show that virus concentrations detected using the PTFE cassette were all higher than those detected using the high-volume sampler. Regarding sampling stress, the face velocity of the high-volume sampler (0.003 m/sec) is actually lower than that of the PTFE cassette (0.3 m/sec). We also observed the same trend in our previous study, where we obtained higher virus concentrations with a PTFE cassette than when with an open-face filter cassette with lower face velocity. Higher concentrations of various inhibitors such as airborne bacteria cosampled in the filters of a high-volume sampler might contribute to the lower sensitivity of this method. The inhibitory rates observed here were also consistent with this hypothesis (Table 2). According to our data, a PTFE cassette is superior for sampling ambient influenza virus.

To date, field study data on airborne influenza virus are extremely limited. Airborne influenza virus has been measured in 4-hr samples in a hospital emergency department at a mean concentration of 6.5 × 10^3^ copies/m^3^ in the study by Blachere et al. (2009). In our previous study, airborne influenza virus concentrations in 4-hr samples were 6.9 × 10^3^ copies/m^3^ and 2.0 × 10^3^ copies/m^3^ in a chicken pen and duck pen, respectively, in a wet poultry market (Chen et al. 2009). The concentration of ambient influenza virus in 24-hr samples measured in our current study was 1–2 orders of magnitude lower than that reported in those two previous studies. Although Blachere et al.’s (2009) study did not specifically mention the detection limit, the lowest positive sample reported was 368 copies/m^3^. According to our previous data, the detection limits using the PTFE cassette coupled with real-time qPCR for influenza A and A/H5 virus were 0.8 copy/m^3^ and 1.23 copies/m^3^, respectively (Chen et al. 2009). In the present study, the lowest concentrations measured in positive samples were 1 copy/m^3^ and 2 copies/m^3^ for influenza A and A/H5 virus, respectively (Table 2). The present study demonstrates that sampling using the PTFE cassette coupled with realtime qPCR is a promising tool for ambient pathogen investigations.

The presence of desert dust in the atmosphere was associated with higher fungal and bacterial CFU concentrations relative to background or clear atmosphere conditions in all previous studies reviewed by Griffin (2007). The culturable bacteria and fungi from air samples were 1 to > 1,500 times higher and 2.1–3 times higher, respectively, when African dust was affecting the region than when it was not (Brown et al. 1935; Choi et al. 1997; Fulton 1966; Griffin et al. 2001, 2003, 2006, 2007; Ho et al. 2005; Kellogg et al. 2004; Kwaasi et al. 1998; Proctor 1935; Prospero et al. 2005; Schlesinger et al. 2006; Wu et al. 2004). During Asian dust events that affect air quality in Taejon, Korea, the average bacterial CFU concentration was 4.3 higher than the concentration observed under normal atmospheric conditions (Choi et al. 1997). In Taiwan, the fungal CFU concentration was 1.01–1.3 times higher during ADS days than during background days (Ho et al. 2005; Wu et al. 2004). These studies demonstrated long-range atmospheric transport of culturable bacteria and culturable fungi in dust storms. Although there are already 14 studies investigating long-range atmospheric transport of culturable bacteria and culturable fungi, transport of viral pathogens using a PCR-based approach with positive detects in dust storms had not to our knowledge been investigated in a previous study (Joo et al. 2002).

For human influenza virus, Hammond et al. (1989) hypothesized that long-range transport of human influenza virus from Asia to the Americas could occur in the winter months because of the low dose of virus required for infection and the prevailing wind patterns over the Pacific (Hammond et al. 1989). Previous virus survival studies report a relationship between particle association/attachment and enhanced survival, thus suggesting that the attachment of infectious viruses to dust particles moving across the ocean might enhance long-range host-to-host transport (Chung and Sobsey 1993; Cox 1995; Labelle and Gerba 1981; Rao et al. 1984). In the present study, we successfully quantified ambient influenza A virus during both ADS days and background days. Our data showed that ambient influenza virus concentration during ADS days was 21 and 31 times higher at the Wan-Li and Shin-Jhuang air monitoring stations, respectively, than that during background days. In addition, we observed all positive samples of A/H5 during episode days at the Wan-Li station. The Wan-Li station is near the northern tip of Taiwan and thus is the first location in Taiwan affected by ADS. In addition, HYSPLIT back-trajectories indicated that ambient influenza virus probably originated from mainland China. According to the OIE (2006) no outbreak was reported in Taiwan during our sampling period, but an outbreak was reported by the People’s Republic of China. Our results indicate that dust storms may be a vector and source of influenza. However, dust from the Gobi desert might not be the only source of ambient influenza virus, based on evidence implicating the contribution of African dust to bacterial and fungal CFU concentrations in ambient air in the Caribbean and the Mediterranean (Polymenakou et al. 2008; Prospero et al. 2005). Actually, airborne influenza virus from any land surface located along the trajectories might contribute to collected samples. More studies are needed to clarify this hypothesis.

Although temperature was significantly lower during episode days than during background days, we observed no significant correlations between climate factors and ambient influenza virus. We observed significant correlations between ambient influenza virus concentration and air pollutants, but only during background days. Similar composition of air pollutants during the ADS period might be the reason. During background days, ambient influenza was significantly negatively correlated with PM_10_ and SO_2_ concentrations at the Wan-Li and Shin-Jhuang stations, respectively. The Wan-Li station is a remote location with little industrial or traffic-related air pollution, whereas the Shin-Jhuang station is located near an industrial center and two highways with heavy traffic. Different profiles of pollutants and climate factors such as temperature and RH at the two stations might explain the differences observed between the Wan-Li and Shin-Jhuang stations. When we pooled data from both stations, ambient influenza concentration was significantly negatively correlated with PM_10_ and PM_2.5_ on background days. Although previous studies have noted a relationship between PM concentrations and enhanced viral survival, we found negative correlations between ambient influenza virus concentrations and PM_10_, SO_2_, and PM_2.5_ (Chung and Sobsey 1993; Cox 1995; Labelle and Gerba 1981; Rao et al. 1984). One possible explanation is that PM_10_, SO_2_, or PM_2.5_ may inhibit the PCR reaction. A second possibility is that PM_10_, SO_2_, or PM_2.5_ might injure airborne virus in ambient air. Previous studies have reported that many compounds in environmental media such as soil and water can inhibit PCR (Alvarez et al. 1995; Jacobsen and Rasmussen 1992), but we are not aware of any study that has specifically examined effects of PM_10_, SO_2_, or PM_2.5_ on viruses.

In summary, we successfully quantified ambient influenza viruses during ADS days and background days. The PTFE cassette used in this study was superior to a high-volume air sampler for ambient influenza virus collection. The concentration of ambient influenza A virus was significantly higher during ADS days than during background days. In addition, A/H5 was detected only during ADS days.

## Figures and Tables

**Table 1:** Primers and probes of influenza A virus and A/H5.

Virus type (target)	Primer or probe	Sequence	Reference
A (*M* gene)	INFA-1	5′-GGACTGCAGCGTAGACGCTT	van Elden et al. 2001
	INFA-2	5′-CATCCTGTTGTATATGAGGCCCAT	
	INFA-3	5′-CATTCTGTTGTATATGAGGCCCAT	
	INFA probe	5′-CTCAGTTATTCTGCTGGTGCACTTGCCA	
A/H5 (*HA* gene)	H5-1	5′-ACGTATGACTATCCACAATACTCAG	Spackman et al. 2002
	H5-2	5′-AGACCAGCTACCATGATTGC	
	H5 probe	5′-TCAACAGTGGCGAGTTCCCTAGCA	

**Table 2:** Comparison of influenza A virus in samples collected for 24 hr using an MFC-PM10 high-volume sampler and a PTFE cassette for both the spiked samples (5 sets, *n* = 10) and unspiked air samples (13 sets, *n* = 26) at the Wan-Li air monitoring station in Taiwan.

	Sampler (mean ± SD)		
Sample type	PTFE cassette	High-volume sampler	*p*-Value^a^
Spiked samples (copies/m^3^)	701.9 ± 309.6	433.1 ± 128.4	0.076
Unspiked samples (copies/m^3^)	76.4 ± 22.09	5.9 ± 8.7	0.606
Inhibitory rate	43%	80%	

a Mann-Whitney *U*-test.

**Table 3:** Ambient influenza A virus and A/H5 on ADS days versus background days at air monitoring stations in Taiwan.

Sampling location/ virus	Measure	ADS days (*n* = 24)	Background days (*n* = 10)	*p*-Value^a^
Wan-Li station				
Influenza A	Positive rate (%)	58 (14/24)	30 (3/10)	0.13
	Mean (copies/m^3^)	268	13	0.02
	Median (copies/m^3^)	135	13	
	Range (copies/m^3^)	1–810	11–15	
	Inhibitory rate (%)	78.6 (11/14)	66.7 (2/3)	

A/H5	Positive rate (%)	13 (3/24)	0 (0/10)	
	Mean (copies/m^3^)	1.8	ND	
	Median (copies/m^3^)	0	ND	
	Range (copies/m^3^)	ND–25	ND	
	Inhibitory rate (%)	100 (3/3)	—	

Shin-Jhuang station				
Influenza A	Positive rate (%)	46 (11/24)	20 (2/10)	0.25
	Mean (copies/m^3^)	276	9	0.11
	Median (copies/m^3^)	89	9	
	Range (copies/m^3^)	4–1,160	5–13	
	Inhibitory rate (%)	72.7 (8/11)	0 (0/2)	

A/H5	Positive rate (%)	0 (0/24)	0 (0/10)	
	Mean (copies/m^3^)	ND	ND	
	Median (copies/m^3^)	ND	ND	
	Range (copies/m^3^)	ND	ND	
	Inhibitory rate (%)	—	—	

ND, not detected (below detection limit).

a Mann-Whitney *U*-test.

**Table 4:** Environmental factors on ADS days versus background days at air monitoring stations in Taiwan.

Sampling location/ environmental factor	ADS days (*n* = 24)			Background days (*n* = 10)			*p*-Value^a^
	Mean	Median	Range	Mean	Median	Range	
Wan-Li station							
PM_10_ (μg/m^3^)	47.71	45.40	0.0 × 10^0^ to 1.5 × 10^2^	32.13	29.15	7.7 × 10^0^ to 6.5 × 10^1^	0.041
PM_2.5_ (μg/m^3^)	31.50	32.21	7.0 × 10^0^ to 7.2 × 10^1^	18.91	14.18	3.5 × 10^0^ to 3.4 × 10^1^	0.005
CO (ppm)	0.35	0.35	2.0 × 10^−1^ to 5.2 × 10^−1^	0.25	0.25	9.0 × 10^−2^ to 3.5 × 10^−1^	0.005
Temperature (°C)	18.47	18.37	1.1 × 10^1^ to 2.6 × 10^1^	24.50	24.69	1.9 × 10^1^ to 3.0 × 10^1^	0.001

Shin-Jhuang station							
PM_10_ (μg/m^3^)	81.55	73.55	1.5 × 10^1^ to 1.9 × 10^2^	49.62	44.17	1.7 × 10^1^ to 1.1 × 10^2^	0.007
PM_2.5_ (μg/m^3^)	46.42	44.11	8.2 × 10^0^ to 9.4 × 10^1^	26.93	25.75	7.0 × 10^0^ to 4.8 × 10^1^	0.008
CO (ppm)	0.88	0.81	4.5 × 10^−1^ to 2.0 × 10^0^	0.59	0.59	3.3 × 10^−1^ to 9.6 × 10^−1^	0.014
Temperature (°C)	18.80	18.07	9.1 × 10^0^ to 2.8 × 10^1^	25.61	26.94	1.7 × 10^1^ to 3.2 × 10^1^	0.002

a Mann-Whitney *U*-test.

**Table 5:** Correlations between ambient influenza A virus and environmental factors during ADS days and background days at air monitoring stations in Taiwan.

Sampling site/ environmental factor	ADS days			Background days		
	*r*	*p*-Value	Sample size (days)	*r*	*p*-Value	Sample size (days)
Wan-Li station						
PM_10_	−0.403	0.051	24	−0.646	0.044	10
PM_2.5_	−0.354	0.090	24	−0.494	0.147	10
NO_x_	−0.189	0.376	24	−0.114	0.754	10
O_3_	−0.012	0.955	24	−0.570	0.086	10
SO_2_	−0.055	0.799	24	−0.114	0.754	10
CO	−0.232	0.275	24	−0.114	0.754	10
Temperature	−0.244	0.250	24	0.038	0.917	10
Rainfall	0.157	0.464	24	−0.325	0.359	10
RH	0.177	0.408	24	0.190	0.599	10

Shin-Jhuang station						
PM_10_	0.066	0.758	24	−0.522	0.122	10
PM_2.5_	0.187	0.381	24	−0.522	0.122	10
NO_x_	0.103	0.633	24	−0.609	0.062	10
O_3_	0.175	0.413	24	−0.261	0.466	10
SO_2_	0.103	0.633	24	−0.696	0.025	10
CO	0.151	0.481	24	−0.522	0.122	10
Temperature	0.006	0.978	24	0.261	0.466	10
Rainfall	0.286	0.176	24	−0.321	0.365	10
RH	−0.260	0.220	24	−0.522	0.122	10

Both stations						
PM_10_	−0.179	0.223	48	−0.591	0.006	20
PM_2.5_	−0.089	0.548	48	−0.571	0.009	20
NO_x_	−0.135	0.359	48	−0.290	0.214	20
O_3_	0.152	0.302	48	−0.370	0.108	20
SO_2_	−0.045	0.761	48	−0.390	0.089	20
CO	−0.113	0.445	48	−0.270	0.249	20
Temperature	−0.104	0.482	48	0.110	0.644	20
Rainfall	0.232	0.112	48	−0.329	0.157	20
RH%	0.039	0.792	48	−0.070	0.769	20

*r*, correlation coefficient.
